# Transcriptome Analysis Identified Genes for Growth and Omega-3/-6 Ratio in Saline Tilapia

**DOI:** 10.3389/fgene.2019.00244

**Published:** 2019-03-20

**Authors:** Grace Lin, Natascha M. Thevasagayam, Z. Y. Wan, B. Q. Ye, Gen Hua Yue

**Affiliations:** ^1^Temasek Life Sciences Laboratory, National University of Singapore, Singapore, Singapore; ^2^School of Biological Sciences, Nanyang Technological University, Singapore, Singapore; ^3^Department of Biological Sciences, National University of Singapore, Singapore, Singapore

**Keywords:** tilapia, growth, meat quality, RNA, gene

## Abstract

Growth and omega-3/-6 ratio are important traits in aquaculture. The mechanisms underlying quick growth and high omega-3/-6 ratio in fish are not fully understood. The consumption of the meat of tilapia suffers a bad reputation due to its low omega-3/-6 ratio. To facilitate the improvement of these traits and to understand more about the mechanisms underlying quick growth and high omega-3/-6 ratio, we conducted transcriptome analysis in the muscle and liver of fast- and slow-growing hybrid saline tilapia generated by crossing Mozambique tilapia and red tilapia. A transcriptome with an average length of 963 bp was generated by using 486.65 million clean 100 bp paired-end reads. A total of 42,699 annotated unique sequences with an average length of 3.4 kb were obtained. Differentially expressed genes (DEGs) in the muscle and liver were identified between fast- and slow-growing tilapia. Pathway analysis classified these genes into many pathways. Ten genes, including *foxK1*, *sparc*, *smad3*, *usp38*, *crot*, *fadps*, *sqlea*, *cyp7b1*, *impa1*, and *gss*, from the DEGs were located within QTL for growth and omega-3, which were previously detected content in tilapia, suggesting that these ten genes could be important candidate genes for growth and omega-3 fatty acid content. Analysis of SNPs in introns 1 and 2 of *foxK1* revealed that the SNPs were significantly associated with growth and omega-3/-6 ratio. This study lays the groundwork for further investigation of the molecular mechanisms underlying the phenotypic variation of these two traits and provides SNPs for selecting these traits at fingerling stage.

## Introduction

Candidate genes are important for understanding the phenotypic variations (Yue, 2014). Candidate gene association studies examine whether the genetic variations in a candidate gene are significantly associated with important traits. Some candidate genes were found to be associated with important traits in aquaculture species (Han et al., 2017; Wang Q. et al., 2017; Wang Y. et al., 2017; Wei et al., 2018). However, the success of candidate gene association studies is heavily dependent on assumptions underlying the selection of genes to be studied, and the number of candidate genes was limited due to the lack of genomic resources in most aquaculture species before 2008 (Yue, 2014). Recently, due to the rapid development of next-generation sequencing technologies (Schuster, 2007), transcriptome analysis (Wang et al., 2009) becomes a powerful tool for identifying candidate genes related to important traits (Qian et al., 2014). RNA-seq is one of the important tools for analysis of transcriptomes (Wang et al., 2009). In fishes, through RNA-seq, many candidate genes have been identified for some important traits (Qian et al., 2014). QTL mapping is another way to identifying genes related to important traits (Collins, 1995; Yue, 2014). By combining transcriptome analysis and QTL mapping, it is possible to identify causative genes underlying important traits (Yue, 2014; Yue and Wang, 2017). The knowledge on the relationship between genotypes and phenotypes will enhance our understanding about phenotypic variations and identify DNA markers for marker-assisted selection (MAS) to accelerate the genetic improvement (Liu, 2008; Yue, 2014).

Tilapia is a common name for over 70 species of fishes that belong to the tilapiine tribe, in the family Cichlidae (Webster and Lim, 2006). Tilapia has become the second most important group of farmed fish in the world with a production of more than 5.3 million tons in 2014 (Food and Agriculture Organization, 2014). The main cultured species globally is the Nile tilapia (*Oreochromis niloticus*). For Nile tilapia, many genetic resources, including genome sequences (Brawand et al., 2014; Xia et al., 2015; Wan et al., 2016) and linkage maps (Kocher et al., 1998; Liu et al., 2013) have been generated in the past 30 years. These resources have been used to map QTL for important traits (Cnaani et al., 2003; Liu et al., 2014; Palaiokostas et al., 2015; Gu et al., 2018), which paved the way for MAS to accelerate genetic improvement. However, the limited sources of freshwater in the world prohibited the expansion of aquaculture of freshwater Nile tilapia. In addition, the low omega-3 content in Nile tilapia is a major health concern of consumption of tilapia (Young, 2009). Mozambique tilapia can adapt to full seawater and produces high omega-3 (Young, 2009), but grows much slower than Nile tilapia (Webster and Lim, 2006). Hybrid tilapia (i.e., saline tilapia) produced by crossing Nile and Mozambique tilapia is able to adapt to full seawater and grows quicker than M. tilapia, but still slower than Nile tilapia (Liu et al., 2013). Therefore, it is essential to increase the growth of saline tilapia to make it economically viable. However, for saline tilapia, genome resources are limited. Not much is known about genes controlling growth and fatty acid contents in saline tilapia.

The aim of this study was to identify candidate genes for growth and fatty acid contents in saline tilapia, by RNA-seq in combination with previous QTL mapping (Liu et al., 2013, 2014; Lin et al., 2016, 2018), to accelerate the genetic improvement of these traits, and to understand more about the molecular mechanism underlying the phenotypic variations in growth and fatty acid contents. We generated transcriptomes in the muscle and liver of fast and slow growing hybrid tilapia, and identified differentially expressed genes located in QTL for growth and omega-3 content (Liu et al., 2014; Lin et al., 2016, 2018). Our study paves the way for detailed analysis the functions of these genes to understand more about the mechanism underlying growth and fatty acids synthesis. We cloned the full-length cDNA of the *foxK1* gene, identified two SNPs in introns 1 and 2 of the gene and found significant associations between the SNPs and growth and omega-3/-6 ratio. Therefore, the SNPs could be useful in selecting superior tilapia at fingerling stage.

## Materials and Methods

### Tilapia, Tissue Samples, and RNA Extraction

At 110 dph, four fast-growing and four slow-growing individuals were randomly selected from the F_2_ family of tilapia, including 522 individuals, used previously for QTL mapping for growth and omega-3 contents (Lin et al., 2018). Briefly, an F_1_ family was generated by crossing one fast-growing red tilapia female and one slow-growing Mozambique tilapia male. An F_2_ family was produced by crossing one F_1_ male and one F_1_ female, which were randomly selected from the F_1_ family. The F_2_ family consisted of 522 offspring and was cultured under normal culture conditions as detailed in our paper (Lin et al., 2018). All F_2_ fish were raised under the same culture conditions and feeding scheme. Fishes were dissected and tissue samples from muscle (M), liver (L), brain (B), gills (G), and intestine (I) were collected and frozen in liquid nitrogen and stored in −80°C for further downstream analyses. Total RNA was extracted using RNeasy^®^ Mini kit (Qiagen, Hilden, German) according to the manufacturer’s instructions. The concentration of the RNA was then determined with Agilent 2100 Bioanalyzer (Agilent, Singapore). A total of 40 total RNA samples (i.e., 4 fast growing fish + 4 slow growing fish × 5 tissues per fish) were generated. For each tissue in each group (fast and slow growing), equal amount of RNA from each of four fish were pooled for cDNA library construction. Hence, there are 10 pooled samples named with M_Fast_, L_Fast_, B_Fast_, G_Fast_, I_Fast_, M_Slow_, L_Slow_, B_Slow_, G_Slow_, and I_Fast_.

### Construction of 10 cDNA Libraries for RNA-Seq

One microgram of total RNA from each pooled sample was used for library construction using TruSeq^TM^ RNA sample prep kit (Illumina, San Diego, CA, United States). In brief, the total RNA was treated with DNase to remove the genomic DNA contamination, and the thus isolated mRNA with poly(A) was enriched using magnetic beads coated with oligo-dT subsequently. The enriched mRNA was fragmented and reverse-transcribed with random hexamer-primers to produce second strand cDNA. The second strand cDNA was purified and ligated with Illumina sequencing index and adapters. Size selection for the cDNA was carried out and enriched to generate the final sequencing libraries. Sequencing of these libraries was then performed on the Illumina HiSeq^TM^ 2000 (Illumina, San Diego, CA, United States) to produce paired end (2 bp × 101 bp) sequence reads. Ten sequencing libraries were constructed.

### Processing of Sequencing Reads and *de novo* Assembly of Reference Transcriptome

The Illumina short raw reads were processed by NGS QC toolkit (Patel and Jain, 2012) to remove adapters and low quality reads (*Q* < 20) and unpaired reads. The filtered high-quality reads were *de novo* assembled using Trinity (version 20140717) (Grabherr et al., 2011; Haas et al., 2013). As Nile tilapia is closely related to the hybrid tilapia in this study, the *de novo* assembly was merged with 45,440 annotated Nile tilapia mRNA sequences from NCBI RefSeq database using CAP3 software (Huang and Madan, 1999) to maximize the chance of presenting as many transcripts as possible. The merged transcriptome was BLASTN-searched against the Nile tilapia mRNA to assign gene descriptions, by using an *e*-value cut-off of 1 × 10^−6^ and selecting the best hit. The reference transcriptome used for subsequent analyses comprised of the set of unique BLASTN-annotated sequences, where a single sequence was selected as a representative for each corresponding Nile tilapia BLASTN match, as well as the remaining unannotated sequences. A BLASTX-search against the NCBI RefSeq protein database was also further carried out for the unannotated sequences to supplement the annotated dataset.

### Identification of Differentially Expressed Transcripts (DETs) in the Muscle and Liver

The filtered reads from the muscle and liver samples (M_Fast_, L_Fast_, M_Slow_, and L_Slow_) were aligned to the reference transcriptome using the CLC Genomics Workbench (version 7.0) “Map reads to reference” tool, with thresholds set at 95% length fraction and 95% similarity fraction, and with default costs for mismatches, insertions and deletions. The BAM files of the mapping were then imported into Partek Genomics Suite (version 6.6) for differential gene expression analysis. Following mRNA quantification to obtain read counts and reads per exon kilobase per million mapped reads (RPKM), differential expression analysis was performed for pair-wise tissues using ANOVA. As there were no replicates in this study, the *p*-value was adapted from the mRNA quantification step where the algorithm estimates a *p*-value by assuming that all the samples are replicated and that the transcripts are evenly distributed across all samples. The *p*-value was subsequently corrected using the conservative Bonferroni method (Noble, 2009; Haynes, 2013) available in the software, to decrease the probability of false positives being detected, correcting for family-wise error rates since pooled samples from the same family are used in this study. The differential expression analysis was first narrowed down to transcripts with BLASTN or BLASTX annotations. Secondly, the global RPKM values were examined to filter off very lowly expressed transcripts, with RPKM >1 set as the minimum threshold for expression (Figure 2). Annotated transcripts were considered differentially expressed if the corrected *p*-value <0.001 and fold change ≥2.

### Functional Classification and Pathway Analysis of DETs

To understand the possible roles of the differentially expressed transcripts (DETs) in conferring the body size of the fish and the lipid content of the meat, the annotation of gene ontology (GO) terms was carried out using Blast2GO (Götz et al., 2008) based on the GO annotations of the corresponding homologs in the NCBI RefSeq database, allowing for an overview of the functional classes. These DETs were then mapped onto KEGG pathways using KAAS webserver (Moriya et al., 2007) to allow us to have an overview of the network of pathways that were involved in conferring the differing body size and lipid content in meat between the fast and slow growing fishes. From the assigned KEGG pathways, 18 and 21 pathways of interest were selected for growth and lipid biosynthesis, respectively.

### qRT-PCR for the Validation of the Expressions of DETs Identified With RNA-Seq

Reverse transcription was carried out using iScript^TM^ reverse transcription supermix (Bio-Rad Laboratories, Hercules, CA, United States). The single strand cDNA was diluted five times and kept in −20°C for further analyses. A total of 21 primer pairs (17 up-regulated and 4 down-regulated) (Supplementary Table S1) were designed using Primer- blast software (Ye et al., 2012) for both muscle and liver transcripts that were differentially expressed between the fast and slow growing fishes. Quantitative PCR (qPCR) was carried out on BioRad iQ5 (Bio-Rad Laboratories, Hercules, United States) using SYBR^®^ Green as fluorescent dye. qPCR reactions were carried out in triplicates for each tissue of four individuals, each 20 μL reaction volume containing 2 μL (10 ng) of 10 times diluted sscDNA, 0.2 μL of each primer of concentration 10 μM, 5 μL of 2× master mix from KAPA SYBR^®^ FAST qPCR Kits (Life Technologies, Carlsbad, CA, United States) and 2.6 μL of sterile water. Raw data was converted to cycle threshold (Ct) values using the software provided by BioRad iQ5 (Bio-Rad Laboratories, Hercules, United States). Quantification of relative gene expression was calculated using the 2^ΔΔct^ method (Livak and Schmittgen, 2001) using beta actin as the housekeeping gene to normalize the relative expression. Paired-t test was used for calculation of *p*-values for comparing the differential expression of genes between fast and slow growing fish. The fold change of each gene was the ratio of expression in fast-growing fish to that in slow-growing fish and all results were compared with RNA sequencing fold change data. The correlation between the data of RNA-seq and qRT-PCR was calculated using Microsoft Excel.

### Mapping DETs in Previously Mapped QTL for Growth and Omega-3 Content

From the QTL identified for growth and fatty acid contents, focus was placed on the three growth traits, body weight (BW), total length (TL), body thickness (BT) and omega-3 fatty acid contents (Lin et al., 2018). In order to locate the genomic regions of the flanking markers where these QTL were mapped, the microsatellite or SNP marker sequences were downloaded from NCBI database and the NGS data from the SNP marker discovery. The genomic locations of the markers and DEGs were identified using the BLASTN program against the Nile tilapia genome (Brawand et al., 2014). DEGs that lie or are within close proximity with the identified QTL regions were selected for further study.

### Identification of SNPs in Introns 1 and 2 of the *foxK1* Gene and Analysis of Their Association With Growth and Omega-3/-6 Ratio

The full-length cDNA of the gene *foxK1* was derived from GenBank (XM_019360121). The genomic DNA sequence was obtained by Blasting cDNA against the whole genome sequence of tilapia in Ensembl Release 91^1^. The exons and introns were identified by aligning the full-length cDNA and genomic DNA sequence using Sequencher v5.0 (GeneCodes, CA, United States). Two pairs of primers (HySNPFoxK1-1FR and HySNPFoxK1-2FR) (Supplementary Table S1) were designed for PCR to identify SNPs in the 5′ UTR-intron 1 and exon 2-intron 2 of the *foxK1* gene. The PCR reactions consisted of the following components: 10 ng of genomic DNA, 1× PCR buffer with 1.5 mM MgCl_2_, 0.2 μM of each primer, 50 μM of each dNTP and 1 unit of Taq DNA Polymerase (Fermentas, PA, United States). PCR was conducted for each individual with the following program: an initial denaturation of 95°C, followed by 34 cycles of 94°C for 30 s, annealing temperature 55–58°C for 30 s, 72°C for 30 s, and a final extension at 72°C for 5 min. After PCR, the PCR products were checked for on 2% agarose gels. The single band PCR product was used for the next step of Sanger sequencing using either a forward or reverse primer with BigDye^®^ Terminator Sequencing Kit (Applied Biosystems, Foster City, CA, United States). The sequencing PCR products were then sequenced using an ABI 3730xl DNA Analyzer (Applied Biosystems, Foster City, CA, United States) following the standard protocol of BigDye sequencing. After sequencing, SNPs were identified using the software Sequencher (GeneCodes, MA, United States). The genotypes at each SNP locus were exported to an excel file for further analysis of their associations with the traits.

The mapping family, containing 522 F_2_ individuals and used for QTL mapping (Lin et al., 2018), was applied for association studies here. The associations between SNPs and traits were determined using ANOVA available in JMP 8.0 software (SAS, NC, United States).

### Analysis the Expression of the *foxK1* Gene Using qRT-PCR

Total RNA from muscle and liver of eight individuals at the age of 110 days post hatch were extracted and reverse transcribed to single strand cDNA, following the instruction of the cDNA synthesis kit (Sigma, CA, United States). One primer pair (FoxK1-F and FoxK1-R) (Supplementary Table S1) was designed using Primer- blast software for qPCR. The qPCR was carried out in triplicates. The expressions of the *foxK1* gene were examined by Quantitative real-time PCR (qRT-PCR) on an iQ5 RT-PCR machine (Bio-Rad, CA, United States). The elongation factor 1-alpha (EF1α) gene (see primers in Supplementary Table S1) was used as an internal control. The cDNA was amplified with the primers. PCR amplification was carried out in a total volume of 20 μl containing 1× Maxima^TM^ SYBR Green qPCR Master Mix (Fermentas, PA, United States), 0.25 μM of each primer and 10 ng template cDNA. The PCR program included a single cycle of 10 min at 95°C followed by 40 cycles of 15 s at 95°C, 30 s at 55°C, and 20 s at 72°C. To confirm the specificity of the amplification, after the completion of the qRT-PCR, a melting-curve analysis was conducted. The expression level of the *foxK1* gene was analyzed using ΔΔCT method (Livak and Schmittgen, 2001).

## Results

### Summary of RNA-Seq

The sequencing of ten libraries from the five tissues of fast- and slow-growing tilapias produced 492,458,402 paired-end (PE) reads of length 100 bp (Table 1). After quality-trimming and filtering of low-quality reads, 486,651,674 high-quality PE trimmed reads (98.88%) were used for *de novo* assembly. In order to obtain a reference transcriptome of saline tilapia, the filtered reads were assembled. Contigs were then merged with 45,440 mRNA sequences of the Nile tilapia available in GenBank. A total of 328,078 contigs with average length of 963 bp and 42,699 annotated unique sequences (average length of 3.4 kb) were obtained. As the aim of the study is to identify candidate genes related with growth and fatty acid contents that are associated with muscle growth and lipid biosynthesis in the liver, detailed analyses were conducted only using muscle and liver transcriptome data.

**Table 1:** Statistics of sequencing reads of 10 libraries generated for saline tilapia using Illumina HiSeq 2000 sequencing.

Name	Type of tissue^∗^	No. of raw reads	No. of reads after trim	Q20 (%)
M_Fast_	Dorsal muscle	42,807,120	42,316,956	98.85
L_Fast_	Liver	57,992,018	57,266,262	98.75
B_Fast_	Brain	38,832,976	38,348,976	98.75
G_Fast_	Gills	49,061,666	48,387,980	98.63
I_Fast_	Intestine	43,791,188	43,192,548	98.63
M_Slow_	Dorsal muscle	42,419,682	41,938,406	98.87
L_Slow_	Liver	69,891,260	69,205,904	99.02
B_Slow_	Brain	44,479,234	43,955,378	98.82
G_Slow_	Gills	62,067,852	61,362,052	98.86
I_Slow_	Intestine	41,115,406	40,677,212	98.93

*^∗^Tissues include dorsal muscle (M) and Liver (L); Subscript Fast – fast growing fish and Slow – slow growing fish.*

### Differentially Expressed Transcripts (DETs) Between the Muscle and Liver of Fast- and Slow-Growing Fish

Annotated transcripts were used in the differential expression analysis. Following a RPKM cutoff of >1, and thresholds for differential expression set as Bonferroni-corrected *p*-value <0.001 and fold change ≥2, 2,236 transcripts in muscle and 3,020 in liver were differentially expressed between the fast and slow-growing fish. 427 and 1,809 transcripts were, respectively, up and downregulated in the muscle, whereas the corresponding numbers for the liver were 1,808 and 1,212 transcripts (Supplementary Table S2). Our data analyses identified 14 DETs that overlapped between muscle and liver datasets with either expressions that agree in both tissues (i.e., both up/down regulated) or contrasting expression (i.e., up regulated in muscle and down regulated in liver) (Supplementary Table S2).

### Functional Classification of DETs

A total of 957 (42.7%) and 1726 (57.2%) DETs were assigned 2088 and 3374 GO terms in the muscle and liver, respectively, covering all three domains: biological process (BP), molecular function (MF) and cellular component (CC). In most of the cases, one transcript was assigned to many terms and these sequences were further characterized into primary subcategories.

**FIGURE 1:**
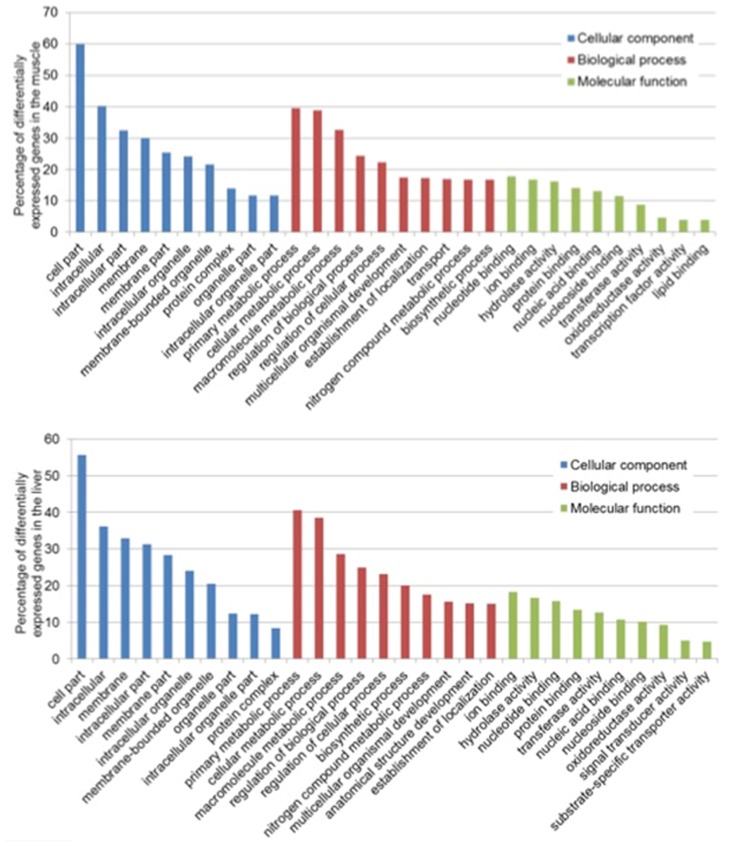
Top 10 functional classification of differentially expressed transcripts in the muscle **(Top)** and liver **(Bottom)** of saline tilapia.

In the muscle, the GO terms with the most assigned genes, in the domain of CC, were cell parts (GO:0044464) with 60 transcripts, intracellular (GO:0005622) with 40 transcripts and intracellular part (GO:0044424) with 34 transcripts. For BP, 40 transcripts were assigned to primary metabolic process (GO:00044238), 39 to cellular metabolic process (GO:0044237), and 34 to macromolecule metabolic process (GO:0043170) and lastly, for MF, 18 transcripts were involved in nucleotide binding (GO:0000166), 16 in ion binding (GO:0043167) and 15 in hydrolase activity (GO:0016787) (Figure 1). In the liver, the most assigned GO terms in the CC domain were cell parts (GO:0044464) with 56 transcripts, intracellular (GO:0005622) with 36 transcripts and membrane (GO:0016020) with 33 transcripts. For BP, 40 transcripts were assigned to primary metabolic process (GO:00044238), 39 to cellular metabolic process (GO:0044237), and 29 to macromolecule metabolic process (GO:0043170) and lastly, for MF, 19 transcripts were involved in nucleotide binding (GO:0000166), 16 in hydrolase activity (GO:0016787) and 15 in ion binding (GO:0043167) (Figure 1).

**FIGURE 2:**
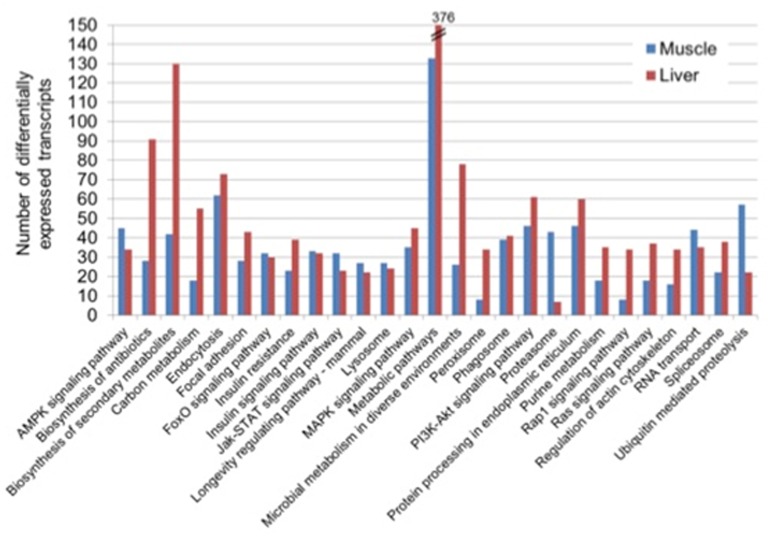
Top 20 KEGG pathways of differentially expressed transcripts in the muscle and liver of saline tilapia.

A total of 844 and 1234 DETs were mapped onto KEGG in the muscle and liver, respectively. In each of the two tissues, the annotation to KEGG via KAAS revealed diverse, but related top five subclass pathways: “metabolic pathways,” “endocytosis,” “ubiquitin mediated proteolysis,” “PI3K-Akt signaling pathway” and “protein processing in the endoplasmic reticulum” in the muscle and, “metabolic pathways,” “biosynthesis of secondary metabolites,” “biosynthesis of antibiotics,” “microbial metabolism in diverse environments,” and “endocytosis” in the liver (Figure 2).

### Validation of DEGs Using qRT-PCR

For the validation of the expression profile of differentially expressed genes (DEGs) in the RNA-sequencing data, primers from 21 DEGs were used for qRT-PCR. The comparison of the fold changes (ratio of RPKM of fast-growing fish to RPKM of slow growing fish) in the RNA-seq and qPCR data showed a significant correlation (*r* = 0.885 *P* < 0.0001) for all the 21 genes tested, indicating that the expression profiling of the DEGs determined by the RNA-seq is reliable and accurate.

### Locating DEGs in Muscle and Liver to Previously Mapped QTL for Growth and Fatty Acids

We focused on the DEGs that are located in QTL for growth and omega-3 contents, which were identified in a previous study (Lin et al., 2018), to facilitate the identification of candidate genes and their polymorphisms that underlie the phenotypic variation of growth and fatty acid content, especially omega-3 fatty acids, in the population. Ten growth and/or lipid biosynthesis related DEGs that were located at reasonably close proximity to the identified QTL for growth (77 kb–1.8 Mb) and omega-3 fatty acid content (<400 kb) (Table 2). For growth related QTL, four genes were found to be located within or close to the QTL region on LG 2, 6 and 7. One of the four genes, *forkhead box K1* (*foxK1*), showed down regulation in fast growing fish and was found to be located approximately 1 Mb downstream of the QTL for both BW and TL on LG 6, while *ubiquitin specific peptidase 38* (*usp38*) was located inside of the detected QTL for BT on the same LG, and was down regulated in fast growing fish. Two other genes, *secreted protein acidic and cysteine rich* (*sparc*) and *smad family member 3* (*smad3*), which were up and down regulated in fast growing fish muscle, respectively, were located approximately 77 and 300 kb downstream of QTL identified for BW and TL, respectively. For omega-3 fatty acid content QTL, three, two and one genes were located within or in close proximity to the QTL regions identified on LG 11, 18, and 20, respectively. On LG 11, for QTL associated with EPA, two genes, peroxisomal *carnitine O-octanoyltransferase-like* (*crot*) located within the QTL region, and *farnesyl pyrophosphate synthase-like* (*fdps*) located 600 kb upstream of the QTL region, were up and down regulated in fast growing fish liver, respectively. As for QTL associated with DHA on LG 11, one gene, *squalene epoxidase* (*sqlea*), located approximately 14 kb upstream of the QTL region, was down regulated in fast growing fish. On LG 18, for QTL associated to ALA and EPA, two genes, *cytochrome P450-family 7-subfamily B-polypeptide 1-like* (*cyp7b1*) and *inositol monophosphatase 1-like* (*impa1*), located within and 140 kb upstream of the QTL region, respectively, and were down and up regulated in fast growing fish liver, respectively. On LG 20, for QTL associated with ALA and EPA as well, one gene, *glutathione synthetase-like* (*gss*), was located approximately 23 kb upstream of the QTL region and was downregulated in fast growing fish liver.

**Table 2:** Ten candidate gene for growth and fatty acids which were identified by combining RNA-seq and QTL mapping, in fast- and slow-growing saline tilapia.

			Associated markers (flanking)			Differentially expressed genes (DEG)			
Traits	LG	Position of identified QTL (cM)^∗^	Name of markers	Genetic position (cM)	Genomic position (bp)	Gene symbol and description	Genomic position (bp)	*p*-value^∗^	Fold change (fast vs. slow)
180BW	2	16.1	66307	10.1	NIL (scaffold)	*sparc*, Secreted protein acidic and cysteine rich	465053-464161	0	2.11
			212092	30.6	386219				
	6	46.5	242870	46.8	14612903	FoxK1, Forkhead box protein K1-like	11140820-11138678	0	−2.59
			242320	46.6	12965663				
180TL	6	46.5	242320	46.6	12965663	FoxK1, Forkhead box protein K1-like	11140820-11138678	0	−2.59
	7	81.3	OMO361	85.0	38380450-38381389	*smad3*, Smad family member 3-like	38064419-38061775	0	−2.72
			259590	77.3	32325128				
180BT	6	36.4	OMO306	37.0	8989979-8991078	*usp38*, Ubiquitin specific peptidase 38	10482767-10481432	0	−8.00
			241939	32.4	11862605				
C20:5n3 (EPA)	11	57.8	85297 85311 84826	57.301 57.805 60.255	16846639 16894308 15514531	*crot*, Carnitine O-octanoyltransferase-like (peroxisomal)	16851102-16850496	0	2.25
						*fdps*, Farnesyl pyrophosphate synthase-like	16149900-16149252	0	−48.33
C22:6n3 (DHA)		46.5	92943 92904	49.781 50.749	7937936 7854666	*sqlea*, Squalene epoxidase a	7872891-7869064	0	−4.56
C18:3n3 (ALA)	18	72.1	160147 160070 98115	69.215 70.272 72.127	26188866 26030795 NIL(scaffold)	*cyp7b1*, Cytochrome P450-family 7-subfamily B-polypeptide 1-like	25673733-25684378	0	−5.00
C20:5n3 (EPA)						*impa1*, Inositol monophosphatase 1-like	26170118-26171402	0	7.68
C18:3n3 (ALA)	20	15.2	184395 183983	15.178 15.991	19773314 18321777	*gss*, Glutathione synthetase-like	19796370-19802128	0	−3.27
C20:5n3 (EPA)									

*^∗^Bonferroni methodology of correction was applied and all differentially expressed genes statistically significant (P < 0.001). ^∗^Regarding to the genomic range, QTL effects, the data can be found in our previous papers (Lin et al., 2016, 2018).*

### Expressions of 10 Candidate Genes in the Muscle and Liver of Fast- and Slow-Growing Saline Tilapia

The expressions (Table 3) of 10 candidate genes (*sparc*, *foxK1*, *smad3*, *usp38*, *crot*, *fdps*, *sqlea*, *cyp7b1*, *impa1*, and *gss*) located in previously mapped QTL for growth or omega-3 content, in muscle and liver of fast and slow growing saline tilapia were examined using qRT-PCR. All the 10 genes showed differential expressions in at least in one tissue between fast- and slow-growing tilapia (Table 3). For example, the expression level of the *foxK1* gene in the liver was much higher in faster-growing tilapia than slower-growing tilapia (16.60 ± 1.16 vs. 1.65 ± 0.48, *P* < 0.001), while the expression level in muscle was higher in slow-growing tilapia than in faster-growing tilapia (5.07 ± 0.69 vs. 1.51 ± 0.15, *P* < 0.05).

**Table 3:** Average expression levels and standard deviations of 10 candidate genes detected by qRT-PCR.

Gene name (symbol)	Muscle (*n* = 3)			Liver (*n* = 3)		
	Slow	Fast	*p*-value	Slow	Fast	*p*-value
*sparc*	1.32 ± 0.19	2.07 ± 0.10	0.27	2.93 ± 3.52	57.15 ± 5.29	0.16
**FoxK1**	**5.07 ± 0.69**	**1.51 ± 0.15**	**0.03**	**1.65 ± 0.48**	**16.60 ± 1.16**	**0.00**
*smad3*	6.06 ± 0.36	1.78 ± 0.18	0.18	7.03 ± 1.10	13.03 ± 2.65	0.53
***usp38***	**17.63 ± 0.09**	**1.88 ± 0.09**	**0.01**	40.89 ± 5.36	1.89 ± 0.99	0.21
*crot*	3.40 ± 3.27	1.48 ± 0.13	0.13	14.78 ± 5.82	94.69 ± 17.19	0.33
***fdps***	**1.62 ± 0.21**	**1.29 ± 0.06**	**0.01**	15.06 ± 3.20	9.07 ± 1.57	0.70
*sqlea*	2.22 ± 0.94	1.35 ± 0.32	0.16	98.25 ± 14.90	9.06 ± 1.10	0.25
*cyp7b1*	1.75 ± 0.31	2.05 ± 0.30	0.67	**16.94 ± 2.42**	**1.46 ± 0.91**	**0.00**
*impa1*	N.a	N.a	−	2.33 ± 0.43	9.47 ± 1.41	0.11
***gss***	**54.71 ± 15.71**	**1.85 ± 0.12**	**0.01**	34.19 ± 3.62	36.63 ± 3.14	0.26

*These 10 genes were identified by combining RNA-seq and QTL mapping, in fast- and slow-growing saline tilapia. One-way ANOVA was conducted on differential gene expression between fast and slow growing fishes. Significant differential gene expression between the fast and slow growing fishes are in bold with p-value <0.05.*

### SNPs in the *foxK1* Gene and Their Associations With Growth and Omega-3 Content

The full-length cDNA sequence of the *foxK1* gene was 4444 bp, including an ORF of 2019 bp encoding 672 amino acids, a 3′ UTR of 1907 bp and a 5′ UTR of 518 bp. The genomic sequence of the gene was 21699 bp, containing nine exons and eight introns. The two SNPs (SNP1 and SNP2, see Supplementary Figure 1) were located in introns 1 and 2 of the gene and inherited together. Therefore, the analysis of the association of one SNP will suffice. ANOVA test showed that there were significant associations between the SNP and body weight, as well as omega-3/-6 ratio (Table 4). The fish with genotype GG grew much quicker than the ones with GC genotype (Body weight at 6 months post hatch: 304.0 ± 69.9 g vs. 250.6 ± 66.9 g, *P* < 0.0001). Similarly, the fish with genotypes CC and GG showed higher omega-3/-6 ratio than those with the genotype GC (omega-3/-6 ratio: 1.58 ± 0.16, 1.68 ± 0.27 vs. 1.48 ± 0.14, *P* < 0.0001).

**Table 4:** Associations between SNPs in the *FoxK1* gene and traits (mean ± SD) in saline tilapia.

SNP genotypes	BW6M (g)^a^	Omega-3 (mg/100 g)^b^	Omega-6 (mg/100 g)^c^	Omega-3/-6 ratio^d^
GG	304.0 ± 69.9	257.1 ± 103.9	164.9 ± 68.6	1.58 ± 0.16
GC	250.6 ± 66.9	271.9 ± 103.5	186.2 ± 76.1	1.48 ± 0.14
CC	287.5 ± 59.5	246.8 ± 118.9	149.9 ± 75.0	1.68 ± 0.27

*^a^P < 0.0001; ^b^P = 0.376 > 0.05; ^c^P = 0.017 < 0.05; and ^d^P < 0.0001.*

## Discussion

In this study, we used RNA-seq in combination with a previous study on QTL mapping (Lin et al., 2018) to identify candidate genes to improve our understanding of the molecular mechanisms underlying growth and lipid biosynthesis in saline tilapia. A reference transcriptome of the hybrid saline tilapia was generated. The number of transcripts (262,282) and average contig size (846 bp) were fairly close to that of the Asian sea bass genome (267,616 contigs, 979 bp) assembled using multiple platforms (Thevasagayam et al., 2015) and that of blunt snout bream (253,439 contigs, 998 bp) (Tran et al., 2015). On the other hand, number of contigs was much higher than those in the Mozambique tilapia (196,178) (Böhne et al., 2014) and the red tilapia (160,762) (Zhu et al., 2016) while the average contig length of 846 bp was higher than that of the Mozambique tilapia(645 bp) (Böhne et al., 2014) and lower than that of the red tilapia (1120.61 bp) (Zhu et al., 2016). This difference of assembly may be due to the difference of species/strains used, sequencing platforms and assembly algorithms and the reference transcriptome.

Global gene expression and variation in transcription activity occurs across the genome and the number and level of transcript isoforms is not always known (Costa et al., 2010; Ozsolak and Milos, 2011; Pelechano et al., 2013). Therefore, in our study, annotation was carried out using nucleotide and protein databases (BLASTN and BLASTX), bringing the number of annotated transcripts to 42,699, which is more than the total number of mRNA transcripts in the NCBI database for both Nile and Mozambique tilapia. This suggests that our reference transcriptome is more comprehensive. Certainly, it is also possible that the hybridization between two species generated some new genes (Liu et al., 2016).

For identifying differentially expressed genes (DEGs) by RNA-seq, different statistical thresholds can be used. By setting a higher statistical threshold, some DEGs may be missed, while by setting a lower threshold, too many genes may be selected. In this study, we set the significance threshold at twofold difference, which is at lower end of the threshold. Therefore, we identified many DETs: 2,236 in the muscle, and 3,020 in the liver. Surprisingly, the majority of the transcripts were down regulated in both tissues in the fast-growing fish. More than 42.7 and 57.2% of the differentially expressed transcripts in the muscle and liver, respectively, were assigned GO. Additionally, the DETs identified for both muscle and liver had diverse cellular functions and were mainly in pathways of metabolic processes, suggesting the complex crosstalk between the muscle and liver in the regulation of metabolic processes for the growth and development of the fish. In this study, the Hippo pathway, which has been reported to promote cell death and differentiation and inhibit cell proliferation (Zhao et al., 2011; Yu and Guan, 2013), showed decreased expression in fast growing fish in our study. As fishes have indeterminate growth, this finding may be suggestive of a positive transcriptional regulation of cell growth in fast growing fishes in our study. However, the crosstalk of the Hippo pathway with other signaling pathways warrants further investigation on how its down-regulation may confer superior growth in our fast-growing fishes. Surprisingly, the mTOR pathway, a positive regulator of growth known to promote anabolic processes, including biosynthesis of proteins, lipids, and organelles and limiting catabolic processes such as autophagy (Laplante and Sabatini, 2009), was generally down-regulated in our fast growing fish. This is in contrast with findings from studies conducted in rainbow trout where slow growing fish had suppressed mTOR signaling (Danzmann et al., 2016). The reason for this discrepancy remains to be further studied.

For the differentially expressed genes (DEGs) in the liver, the general trend for fatty acid (FA) content associated pathways, such as the biosynthesis of unsaturated FA, FA elongation, alpha linolenic acid and linolenic acid metabolism, is up-regulation in the fast-growing fish. This is in contrast to salinity challenged tilapia, where, it was reported that osmotic stress decreases these FA pathways in order to cope with stress (Xu et al., 2015). In addition, the main catalyst of FA degradation, carnitine O-palmitoyltransferase 2 (*cpt2*), which was previously found to be affected by dietary changes in Atlantic salmon and predominates in red muscle(Frøyland et al., 1998), was down-regulated slightly. To date, there have not been many studies on function of *cpt2* in lipid biosynthesis in the liver in fishes, however, accelerated growth has been reported in European sea bass (Santulli and D’Amelio, 1986) and African catfish (Torreele et al., 2007) that have been fed with a diet supplemented with carnitine. Genes in the (peroxisome proliferators-activated receptors) PPAR signaling pathway such as fatty acid desaturase 2 (*fads2*), retinoic acid receptor (*rxr*) and fatty acid binding proteins (*fabp*), known to play a major role in lipogenesis and the biosynthesis of fatty acids (Nakamura et al., 2004), were generally up regulated. We think that this could most probably explain the higher fatty acid content in fast-growing saline tilapia in our study since lipogenesis and biosynthesis of fatty acids are up-regulated in these fish. In order to improve our understanding of the pathways and genes associated with growth and biosynthesis of fatty acids in saline tilapia, further enrichment analyses using GSEA (Subramanian et al., 2005) and DAVID (Huang et al., 2008) should be conducted using the well annotated and assembled tilapia genome.

In this study, ten DEGs from the muscle and liver were located inside or within close proximity of QTL regions for growth and fatty acid content, identified in our previous studies (Liu et al., 2014; Lin et al., 2016, 2018). The 10 candidate genes in QTL are *sparc*, *foxK1*, *smad3*, *usp38*, *crot*, *fdps*, *sqlea*, *cyp7b1*, *impa1*, and *gss*. Although there is some information about their functions in model organisms and humans, not much information about their role in growth and fatty acid synthesis and storage in food fish is available. Therefore, it is essential to study their functions in food fish. In this study, we selected the *foxK1* gene from the 10 candidate genes to study some of its functions and found that the *foxK1* gene was significantly differentially expressed in both muscle and liver. Previous studies in mice reported that *foxK1* promotes cell proliferation through (i) translational repression of forkhead box protein O4 (*foxo4*); (ii) inhibiting myocyte differentiation through myocyte enhancer factor 2 (*mef2*) (Shi et al., 2012); (iii) repressed autophagy of muscle cells through the restriction of acetylation of histone H4; and (iv) expression of critical autophagy genes (Bowman et al., 2014). It is interesting to note that the expression of *foxK1*-*alpha* agreed with the differential gene expression in the muscle between fast and slow growing fish in this study. Our association study showed that the SNPs in the gene were significantly associated with growth and omega-3/-6 ratio, suggesting the SNPs may be useful in selecting fish, which grow fast and produce a higher omega-3/-6 ratio in fingerlings. However, it is not known whether the association is due to the function of the gene or/and a linked QTL for these traits. Further studies on the role, in the liver of fishes, pertaining to lipid biosynthesis would be interesting since it was observed to be up regulated in the liver of fast-growing fish in our study. CRISPR-Cas 9 technology, a novel technology for genome editing (Sander and Joung, 2014), could be used to knockout these genes to investigate their roles in conferring phenotypic variation in the saline tilapia.

## Conclusion

By combining the analysis of transcriptomes in the liver and muscle of fast- and slow-growing saline tilapia conducted here with previous studies on QTL mapping for growth and omega-3 contents (Lin et al., 2018; Liu et al., 2014; Lin et al., 2016), we identified 10 candidate genes for these traits. An association study confirmed that two SNPs in intron 2 of one of these candidate genes *foxK1* was significantly associated with growth and omega-3/-6 ratio. The SNPs associated with these traits may be useful in MAS to accelerate the genetic improvement of these traits. The transcriptomes and the 10 candidate genes supply an important resource for further understanding the molecular mechanisms underlying phenotypic variations.

## Data Availability

The raw reads generated for this study have been deposited in BioProject Accession: PRJDB7318. The accession numbers for the RNA-seq data are SAMD00137288 (Big Brains), SAMD00137287 (Big Gills), SAMD00137286 (Big Intestine), SAMD00137285 (Big Liver), SAMD00137284 (Big Muscle), SAMD00137283 (Small Brains), SAMD00137282 (Small Gills), SAMD00137281 (Small Intestine), SAMD00137280 (Small Liver), and SAMD00137279 (Small Muscle).

## Ethics Statement

All handling of tilapia was conducted in accordance with the guidelines on the care and use of animals for scientific purposes set up by the Institutional Animal Care and Use Committee (IACUC) of the Temasek Life Sciences Laboratory, Singapore. The IACUC has approved this study within the project “Breeding of Tilapia” [approval number TLL (F)-12-004].

## Author Contributions

GY initiated and coordinated the research project for GL. GL conceived and conducted the analysis. GL and GY drafted and finalized the manuscript. NT, ZW, and BY assisted with experiments, data analysis, and manuscript preparation.

## Conflict of Interest Statement

The authors declare that the research was conducted in the absence of any commercial or financial relationships that could be construed as a potential conflict of interest. The reviewer JHX declared a past co-authorship with one of the authors GY to the handling Editor.

## Footnotes

1 https://asia.ensembl.org/index.html

## Supplementary Material

FIGURE S1 Two SNPs (SNP1 and SNP2) identified in FoxK1 gene of saline tilapia.

TABLE S1 Primers used in the current study.

TABLE S2 Differentially expressed transcripts in the muscle and liver, respectively, between fast- and slow-growing tilapia.
