# Supplementary figures and images for: Transcriptome Analysis Identified Genes for Growth and Omega-3/-6 Ratio in Saline Tilapia
Source: Front Genet. 2019 Mar 20;10:244. doi: 10.3389/fgene.2019.00244 (PMC6435965; doi:10.3389/fgene.2019.00244)

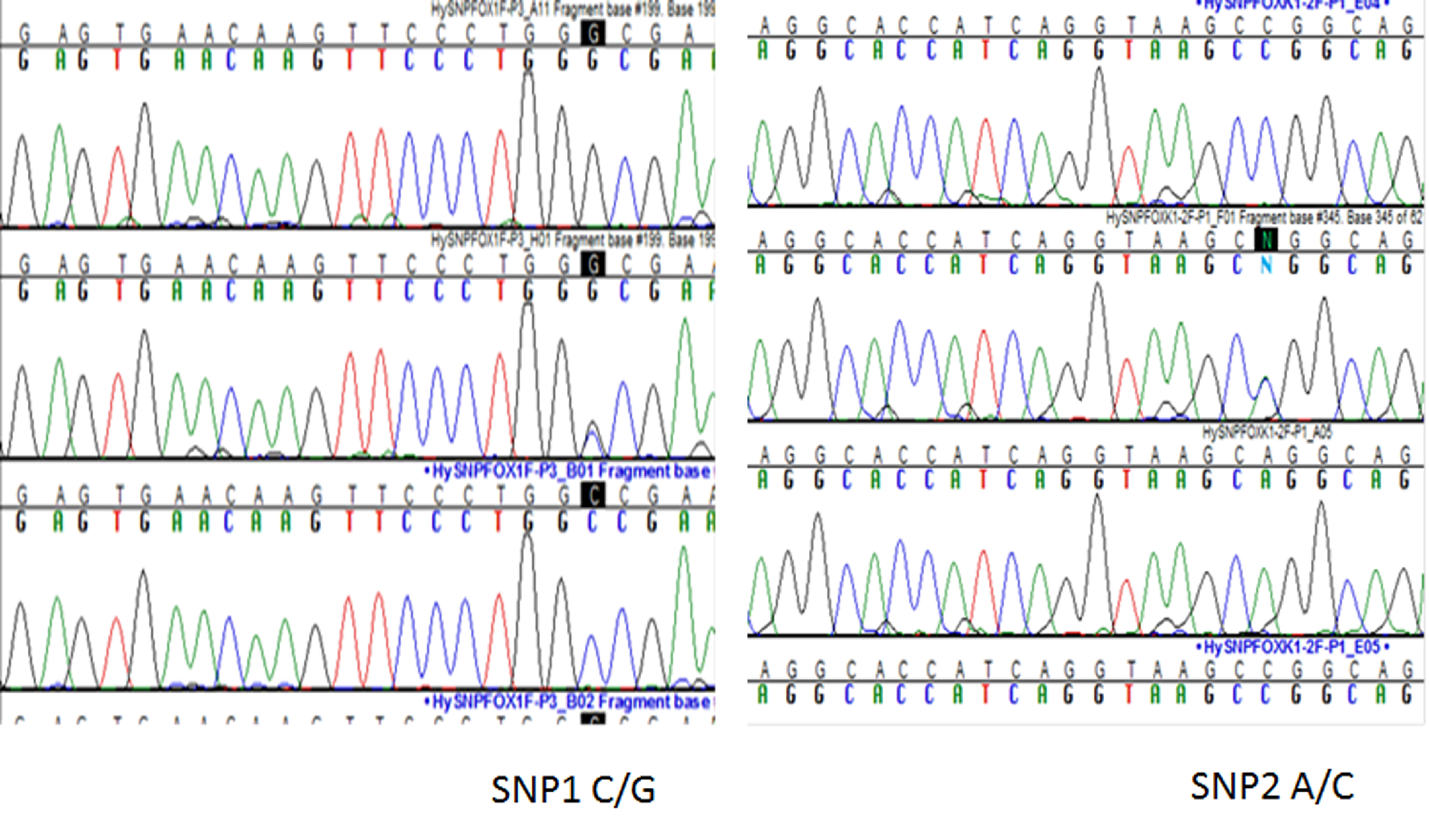

Supplement: FIGURE S1 — Two SNPs (SNP1 and SNP2) identified in FoxK1 gene of saline tilapia. [file Image_1.JPEG]
