# Supplementary material for: Transcriptome Analysis Identified Genes for Growth and Omega-3/-6 Ratio in Saline Tilapia
Source: Front Genet. 2019 Mar 20;10:244. doi: 10.3389/fgene.2019.00244 (PMC6435965; doi:10.3389/fgene.2019.00244)
Supplement: TABLE S1 — Primers used in the current study. [file Table_1.docx]

Supplementary Table 1. Primers used in the current study

A. List of 21 pairs of primers used for real time qPCR analyses of gene transcript analysis for the verification of differentially expressed genes in the muscle and liver of salt tolerant tilapia.

|  | | | |
| --- | --- | --- | --- |
|  | Sequence (5'->3') | | |
| Primer Name | Forward | | Reverse |
| NTsmpxv2_1 | | TTGTTTCATCGACTGGTCAGCGG | TGGACGCAGAGCTCCCATCG |
| NTmyoz_1 | | ACCCCTGAGCCTCCGTGTCT | ACGTAACCGCTGGTACCCTGG |
| NTtmod1v4_10 | | CGGGCACTTGGCTGCCTTTG | ACAGCACACCTCAGCAACCCC |
| NTmyoD1_8 | | AAACGGCGGTCTGAAGAGCGAA | GCTTCACCCACCGGCACAGTT |
| NTnrap_3 | | TCAGCAGTGCAGTGAGAGGCTG | CTTGCTCCCCAGACGCCTTGT |
| NTtgfb3_5 | | CGTGGGTCGCTCCCCGAAAG | CCCATGCCTTGCTTCTCACAGGG |
| NTgadd45g_2 | | CGTCAGAGGGGAACAAGTCAGGT | CTGCATTGCTCTGTCTGCATGTGAA |
| NTcas3_8 | | CCCAGCAGCAGGCCCTTTAACC | GCAACACACTCAGGCCCGGAAAT |
| NTfoxo3_7 | | GAGCCCCAAAAGCGGCCAAG | GCCGCGCCAGAGCCATTG |
| NTcacnb1_10 | | GGTGGATCGGGCGTCTGGTG | GCCGGGCCACCTGTTCCAG |
| NTlpin2_4 | | AGGCTGGAGCTGACGCTCTGT | GGTGTCCGCCAGGAACCAAGATG |
| NTfabp10a_6 | | TTGAGGGCGGCAAACTGGTC | CGACCCAATGGTCATAGTCTCAACC |
| NTadipoR2_1 | | CAGCCAGACCACGGACAGACC | GGGAGCGGACAGACTTGCGG |
| NTpcdb1_5 | | CTGGTCCATCATCACATGACGTGC | ATACGGTGTAGACCATGGGTGTCG |
| NTscd_5 | | ATCGGCTAGTCTCCAGGGGTGG | GCCTGTCGCCTCTAGCTCGG |
| NTrxrgamma_9 | | CCGCCTCTTTTGCTCTTCACGG | CTTCGGGCGCAAATGGTAGCG |
| NThmgcs1_10 | | TGCACAGTGGCAACGAGAGGG | CGAGGCCTGTGAAGGGTCCG |
| NTfaxdc2_4 | | GTTTTCCGGGCTTGGGTCAGC | GCTTCCCCCAGCTTCCCTCG |
| NTabhd5_1 | | CGCCCTCTGGGCTCTCAACC | CACTCCTCCAGGGCTGCCAC |
| NTlss_3 | | CGGAGGGTTTGACTGATCTGACAGC | GGTCTGTGGCTGGCTCCGTC |
| NTelovl6_4 | | TGCTGTGGTCACTCACCCTTGC | AGTGTCACCCAGCTCTGGTGCTT |

B. Two pairs of primer pairs for association studies of FoxK1 to growth and fatty acid content.

| Primer | | Sequence (5’->3’) | | Product length (bp) |
| --- | --- | --- | --- | --- |
|  |  | Forward | Reverse |  |
| HySNPFoxK1-1 | TAC TTT AAG GCA CGC CAA CAT AGT | | AAG TAA CCG CTA GAC CTC CAC TGT | 843 |
| HySNPFoxK1-2 | ACC ATT TCC CCC TGC AAT ACA AGT | | ACT CTG CAT CTC AGG GGT GTT CAG | 749 |

C. A List of primers used for gene expression studies of candidate genes in muscle and liver of fast and slow- growing saline tilapia.

|  | Sequence (5'->3') | | |
| --- | --- | --- | --- |
| Primer | Forward | | Reverse |
| Sparc | ACC GAA GTG GGG GCC AAT CC | GTG GAG GGG TCC TGG CAC AC | |
| FoxK1 | CGG GGT CAT CGG GGT ATC GC | ACT CCG CTC GCT GCT CAC TC | |
| Smad3 | GAG GCA GTC TAC CAA CTC ACC AGG | CAC TAG AGC AGT GGA TGC TGG GG | |
| Usp38 | TCT TGG AAG CGT CTC GGC CC | GTG TCG GCT GGG GAG ACA GC | |
| Crot | TCT GCC GGA GCA GAC CTT TCA G | CAG AGG CGA ACG GAC GAA CTG | |
| Fdps | CCC GCC CTC CCA GCT TAC C | GGG TTG CCC TCT CCG AGT CA | |
| Sqle | CGC CAT GGC CGA GCC AAA TG | ATG CGA GCC TTC CCG GAC AC | |
| Cyp7b1 | TGC AGT TCG TCC GTC AGG AGC | ATG GAG GAC GAG CAC AGG CG | |
| Impa1 | GGT GGT GAC AGA GAT CGG GGC | CAT GTC AAC CGC TGC TGT GCC | |
| Gss | CAA TGG CCT TGG CAG CAC CAG | AGG GGC TGC CTT GTC TCA TCA AG | |
